# Solar water disinfection in large-volume containers: from the laboratory to the field. A case study in Tigray, Ethiopia

**DOI:** 10.1038/s41598-022-23709-5

**Published:** 2022-11-07

**Authors:** Ángela García-Gil, Rafael A. García-Muñoz, Azahara Martínez-García, Maria Inmaculada Polo-López, Araya Gebreyesus Wasihun, Mekonen Teferi, Tsehaye Asmelash, Ronan Conroy, Kevin G. McGuigan, Javier Marugán

**Affiliations:** 1 Department of Chemical and Environmental Technology (ESCET), Universidad Rey Juan Carlos, C/Tulipán s/n, Móstoles, 28933 Madrid, Spain; 2 Plataforma Solar de Almería-CIEMAT, Carretera Senes, Km 4, 04200 Tabernas (Almería), Spain; 3 Department of Medical Microbiology and Immunology, College of Health Sciences, Mekelle University, Tigray, Ethiopia; 4 Department of Biology, College of Natural and Computational Sciences, Mekelle University, Tigray, Ethiopia; 5 Department of Medical Microbiology, College of Health Sciences, Aksum University, Tigray, Ethiopia; 6 Royal College of Surgeons in Ireland (RCSI), Data Science Centre, Dublin 2, Ireland; 7 Department of Physiology and Medical Physics, Royal College of Surgeons in Ireland (RCSI), Dublin 2, Ireland

**Keywords:** Chemical engineering, Environmental sciences, Diarrhoea

## Abstract

The lack of safe drinking water affects communities in low-to-medium-income countries most. This barrier can be overcome by using sustainable point-of-use water treatments. Solar energy has been used to disinfect water for decades, and several efforts have been made to optimise the standard procedure of solar water disinfection (SODIS process). However, the Health Impact Assessment of implementing advanced technologies in the field is also a critical step in evaluating the success of the optimisation. This work reports a sustainable scaling-up of SODIS from standard 2 L bottles to 25 L transparent jerrycans (TJC) and a 12-month field implementation in four sites of Tigray in Ethiopia, where 80.5% of the population lives without reliable access to safe drinking water and whose initial baseline average rate of diarrhoeal disease in children under 5 years was 13.5%. The UVA dose required for 3-log reduction of *E. coli* was always lower than the minimum UVA daily dose received in Tigray (9411 ± 55 Wh/m^2^). Results confirmed a similar decrease in cases of diarrhoea in children in the implementation (25 L PET TJC) and control (2 L PET bottles) groups, supporting the feasibility of increasing the volume of the SODIS water containers to produce safer drinking water with a sustainable and user-friendly process.

## Introduction

Water is essential for life, and ensuring its accessibility, on-site availability, and the use of improved water sources free from faecal and priority chemical contamination are some of the biggest challenges that humanity is currently facing. The United Nations included the specific 6th Sustainable Development Goal on safe water and sanitation in the 2030 Agenda for the Sustainable Development^1^ since water accessibility is not guaranteed for 29% of the global population. In addition, water availability is becoming more unpredictable and problematic due to global warming^2–4^. Global population growth, the increase in economic activities that consume large amounts of water, and urbanisation also contribute to the increase in demand for water^5,6^.

The lack of safe drinking water impacts upon low-income nations more than others^7^. To deal with this situation, user-friendly, sustainable, and low-cost household water treatments are required. Boiling, chlorination, filtration, and flocculation are common examples of household water treatments with estimated costs of $0.66 per person per year for chlorination, $3.03 for filtration and, $4.95 for flocculation derived from reagents and replacements^8^.

**Figure 1:**
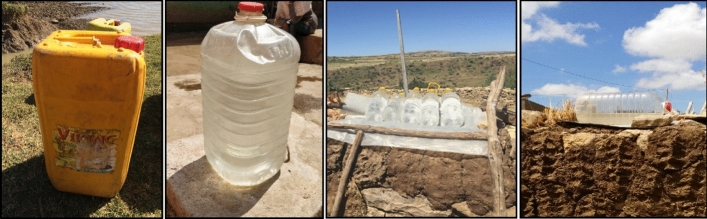
From left to right: Photographs of the standard opaque 25 L jerrycan used in Sub-Saharan Africa and 25 L PET TJC used for solar water disinfection and photographs of the 25 L PET TJC and 2 L PET bottles exposure on site in households of Tigray Region in Ethiopia.

Solar water disinfection (SODIS) is another household water treatment based on the combined effect of UV irradiance and elevated water temperature to inactivate pathogens^9,10^. Its use was pioneered by Acra and colleagues in the 1980s^11^. The most widely accepted procedure for this simple technology is described in detail in the “*SODIS manual: Guidance on solar water disinfection*” published by Luzi et al. (2016)^12^. Briefly, water with a maximum turbidity level of 30 Nephelometric Turbidity Unit (NTU) should be exposed to the sunlight in polyethylene terephthalate (PET) bottles of 2 L for 6 h on sunny days and 48 h on cloudy days to achieve the complete disinfection. This process has been accepted by the World Health Organisation (WHO) and has been recommended for low-to-middle-income countries and natural disasters^13,14^. Many successful studies have reported SODIS implementation under diverse field conditions in different locations such as Kenya, Cameroon, Latin America, and Asia^15–19^. Indeed, even in water with a turbidity level higher than 30 NTU, SODIS offers significant reduction in disease risk^20^. One major advantage of SODIS is that it simply uses plastic bottles, making SODIS one of the cheapest household water treatments, with an estimated cost of $0.63 per person per year^8^. The limitation of using plastic bottles is that using 2 L PET bottles would require treatment of several bottles per person to meet the daily water demand. SODIS use also requires changes in social behaviour that sometimes generate obstacles to uptake. To improve the likelihood of uptake and continued use, SODIS systems should be user-friendly, supported by local members, and ergonomically designed^9,10^. In addition, habits, lifestyles and traditions should be taken into account to improve the acceptability of the systems by the population. For example, in Ethiopia and across most areas of sub-Saharan Africa, 20–25 L jerrycans are commonly used for water collection and transport. Standard jerrycans are typically made of yellow opaque large-density polyethylene (HDPE) (Fig. 1). Consequently, solar radiation cannot be transmitted through the container material, rendering solar disinfection impossible. On this context, the use of 25 L PET transparent jerrycans (TJC) to implement solar disinfection may increase social acceptance (Fig. 1).

This work describes a pilot study of large-volume SODIS in the field using 25 L PET TJC. Laboratory evaluation under controlled conditions was used to simulate field conditions, followed by microbiological evaluation under real sunlight, and finally a Health Impact Assessment (HIA) in the field in rural communities in the Tigray region of Northern Ethiopia, where previous studies showed the significant prevalence of diarrhoea^21^, anaemia and internal parasitosis^22^ among pre-school children. The goal of this study was to evaluate the scaling up of the SODIS process by comparing the efficacy of the 25 L PET TJC with that of 2 L PET bottles in both water disinfection and disease prevention. As a final validation, diarrhoeal episodes in children under 5 years old were monitored in the field by comparing communities using the 25 L PET TJC with a control group using standard 2 L PET SODIS bottles.

## Results

### Microbiological evaluation of the transparent jerrycans

Most populations lacking safe drinking water are located in regions of the world with the highest exposure to sunlight^23,24^. To ensure the feasibility of the SODIS process, the UV daily dose must be higher than the required UV dose to inactivate the microbial population in water. Therefore, the daily accumulated values of total incident radiation and temperature during the year in Tigray, Ethiopia, were obtained using a solar calculator tool^25^. Results of the simulation in Tigray, Ethiopia, are shown in Fig. 1. Tigray’s climate provides a relatively constant irradiance throughout the year, with the major variations resulting primarily from differences in cloud-cover between the wet and the dry seasons. The heaviest rainfalls occur in June, July and August; therefore, these three months correspond to the period with lowest accumulated dose per day. Based on recorded historical data (over the period 2007–2019), the month with the lowest average value is August, with a daily accumulated dose of 9411 ± 55 Wh/m^2^. The solar spectra for solstices in Tigray were also calculated using the solar calculator^25^ (Fig. S1.A). Assuming an average of 4.7% of UVA radiation in the total solar spectrum (Table S1), the minimum accumulated UVA dose during the year in Tigray region is 442.4 ± 2.6 Wh/m^2^. Only UVA radiation is considered since PET does not transmit UVB radiation^26^.

Once the minimum UVA daily dose in Tigray is known, experiments under controlled conditions were performed to determine a first approximation of the expected required UVA doses in real conditions. Firstly, the direct relation between the disinfection rate and the irradiance value was assessed with experiments at different UVA irradiance values (16.6, 21.6, and 28.2 W/m^2^) maintained at a water temperature of 25 °C (low enough to avoid any possible thermal inactivation) and in plain water (to avoid any interference with naturally occurring substances in water). The UVA dose and the time to achieve 3 log reduction viable (LRV) of *E. coli* can be found in Fig. 3 (reference experiment at 16.6 W/m^2^ in dark blue, and at 21.6 and 28.2 W/m^2^ in yellow). The times required to achieve this reduction vary (from 1.7 to 2.8 h), however, the corresponding accumulated doses calculated reveal very similar values (46.8–47.0 Wh/m^2^ UVA dose), confirming that the disinfection rate is proportional to the irradiance. In addition, the required UV dose took a value almost ten times lower than the minimum daily accumulated radiation in Tigray (442.4 ± 2.6 Wh/m^2^), achieved in less than 3 h, indicating it is possible to produce safe drinking water using SODIS in large-volume containers when low turbidity water (< 5 NTU) is used.

The effect of water temperature on the disinfection rate was also studied. The initial temperatures selected were 25 °C (reference experiment), 35 °C, and 45 °C, with the UVA irradiance set at 28.2 W/m^2^ in plain tap water. Data from the required UVA dose and time to achieve 3-LRV are shown in Fig. 3. As can be observed, the higher the temperature, the lower the required time to achieve 3 LRV, and also the lower the required dose. In addition, when the temperature rises to 35 °C and 45 °C, the required UVA dose is halved and reduced by 70%, respectively (47.0 Wh/m^2^ for 25 °C, 23.5 Wh/m^2^ for 35 °C, and 14.1 Wh/m^2^ for 45 °C). This dramatic effect is explained by the synergistic effect between irradiance and temperature^27–29^. Water temperatures during solar exposure easily achieve the 35 °C which ensures the water disinfection.

Finally, the presence of naturally occurring substances in water was evaluated: transparent (TDOM) and coloured (CDOM) dissolved organic matter, (bi)carbonates (CO_3_^2−^/HCO_3_^−^) and solids measured as turbidity. According to SODIS Guidance^12^, maximum levels of 30 NTU of turbidity are recommended. In this study, turbidity up to 50 NTU has been studied to ensure disinfection under rainy season conditions in which surface water can have a heavy particulate load. In practice, turbidity can decrease during solar exposure through sedimentation. The UVA dose and the time to achieve 3-LRV can be found in Fig. 3 and their corresponding inactivation profiles can be found in Figs. S2A–C and S3A–B. As can be seen, similar doses are required to achieve 3 LRV in plain water (reference experiment) as in water with bicarbonates or TDOM. In contrast, higher doses are needed for turbid water (solids) or CDOM. However, all the required doses remain below the minimum accumulated UVA dose observed in Tigray (442.4 ± 2.6 Wh/m^2^), even far below those for normal water composition (turbidity lower than 30 NTU).

**Figure 2:**
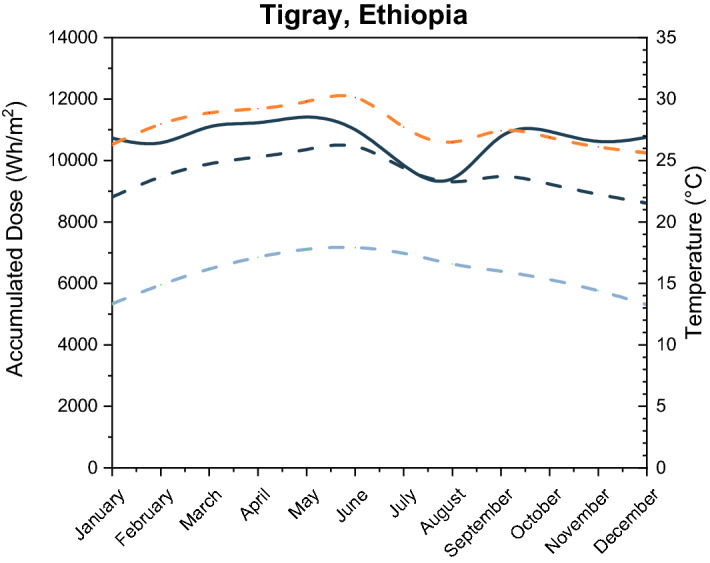
Accumulated global dose of solar light in the Tigray region along the year (solid line: accumulated global dose, dash lines: maximum (orange), average (black) and minimum (blue) temperatures).

Based on these promising laboratory results, a second set of experiments was performed under real sunlight conditions in Almería (Spain) where Summer temperature and irradiance conditions are similar to Tigray (see Fig. S2.B from June to August compared with Fig. 2).

**Figure 3:**
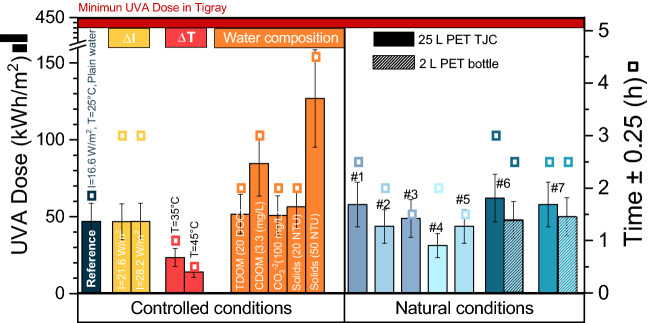
UVA dose (bars) and time (squares) to achieve 3-LRV in SODIS containers under controlled conditions (yellow: UVA irradiance (I), red: temperature (T), orange: water composition) and different natural conditions: well water and #1: $\overline{\mathrm{I} }$= 23.2 W/m^2^, T = 23–27 °C; #2: $\overline{\mathrm{I} }$= 21.9 W/m^2^, T = 26–32 °C; #3: $\overline{\mathrm{I} }$= 39.1 W/m^2^, T = 29–33 °C; #4: $\overline{\mathrm{I} }$= 15.7 W/m^2^, T = 40–41 °C; #5: $\overline{\mathrm{I} }$= 29.2 W/m^2^, T = 40–42 °C; #6: $\overline{\mathrm{I} }$= 20.7 W/m^2^, T = 23–26 °C; and #7: $\overline{\mathrm{I} }$= 23.2 W/m^2^, T = 17–31 °C.

Combinations of different irradiance and water temperature values were tested to study the effect of weather fluctuations. For the four experiments with an initial temperature of around 25 °C (experiments #1, #2, #3, and #6)), the UVA doses required to achieve 3-LRV were between 43.7 and 62.1 Wh/m^2^ (Fig. 3). Although these values are not very precise —since two different bacterial strains (wild and K12 *E. coli*) and different water matrices (neutralised tap water and well water) were used for experiments under controlled and natural conditions, respectively—they are close to those used for controlled conditions (around 47.0 Wh/m^2^) and are below the minimum accumulated dose in Tigray for a day (442.4 ± 2.6 Wh/m^2^). In addition, when the day is sunny and hot (experiments on a sunny day around 40 °C of initial temperatures), the UVA global dose required to achieve 3-LRV significantly drops. Taking into account the similar irradiance conditions, the fact that in all the studied scenarios complete disinfection was achieved in less than 3 h confirms the feasibility of the process.

In addition, to compare the requirements of the process depending on scale-up, simultaneous experiments were performed in two different volume containers: the common 2 L PET bottles of standard SODIS process and in the 25 L PET TJC. In order to study only the effect of the volume, the initial water temperature was set at around 25 °C (Fig. 3, #6 and #7). As can be observed, the higher the irradiance level, the lower the exposure time to achieve 3-LRV (2.5 h for #7 ($\overline{\mathrm{I} }$= 23.2 W/m^2^) vs 3 h for #6 ($\overline{\mathrm{I} }$= 20.7 W/m^2^)). However, for the same type of container, the required dose is practically the same, independent of the irradiance, confirming the findings of the study under controlled conditions. Additionally, as might be expected, the required UVA dose for large-volume containers was higher than for small-volume containers, exactly 1.2 times higher (~ 60 Wh/m^2^ vs ~ 50 Wh/m^2^). However, the daily dose in Ethiopia easily exceeds the 60 Wh/m^2^, and the increase of 1.2 times the dose is acceptable in light of the 12.5 times higher volume (25 L vs 2 L) of disinfected water produced.

### Health impact assessment in Ethiopia rural population

**Figure 4:**
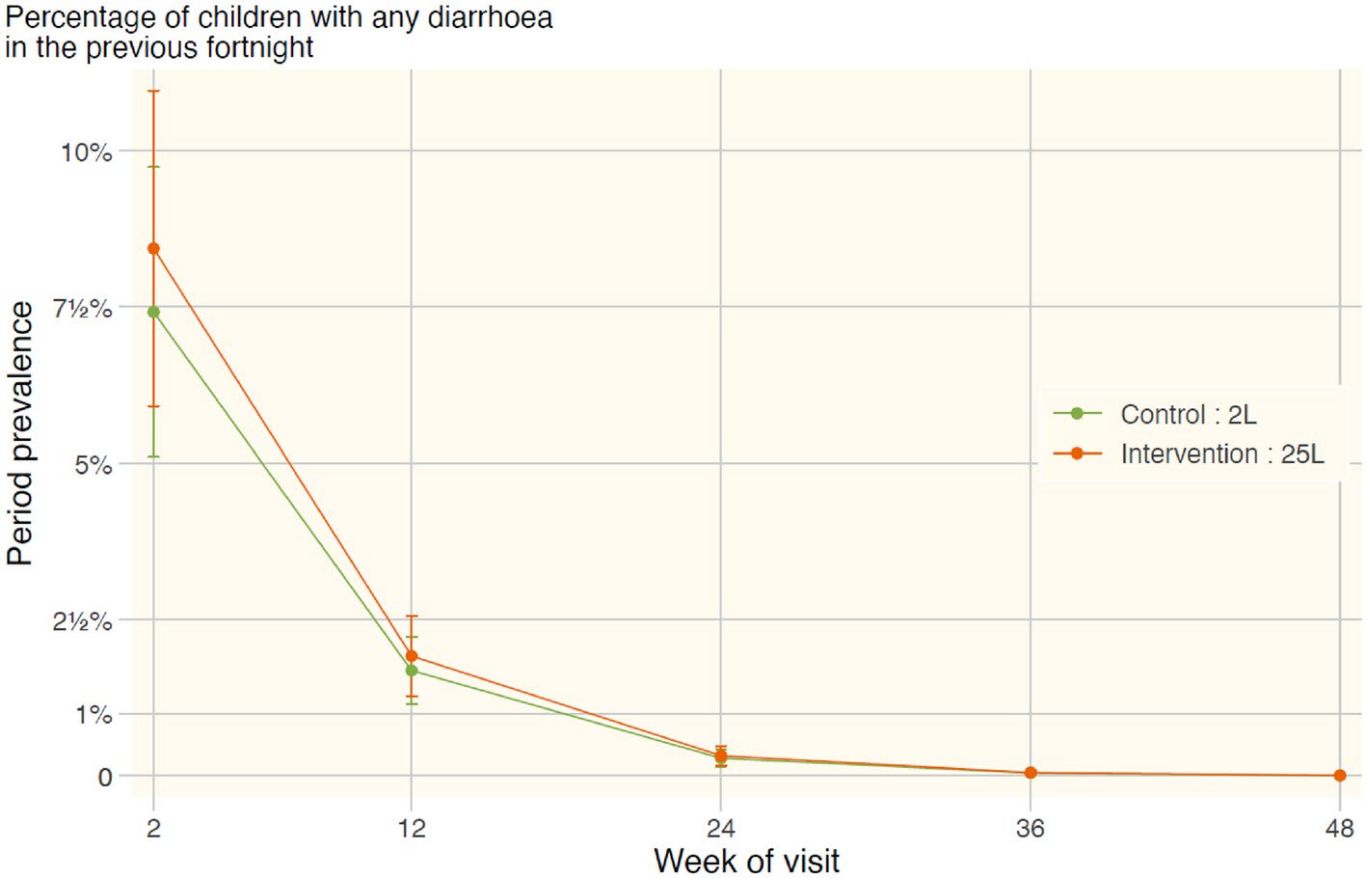
Evolution of the percentage of children with any diarrhoea in the previous fortnight.

To confirm the feasibility of this sustainable scaling-up of the SODIS process, microbiological results should be corroborated with the corresponding health impact on the population at risk. 1147 children were recruited into the field study, of whom 513 (45%) were in households using the 25 L PET TJCs. The number of children recorded at each visit to the sites remained quite stable in Tsawnet and Harena, however, they declined in Dengolat and Grat-Tsatse (all of them are rural communities in Northern Ethiopia in the Tigray region). By week 48, the last fortnightly follow-up visit, there were 930 children under follow-up, of whom 48% were in households using 25 L PET TJCs. In three of the four areas, the proportion of children in the intervention group (using the 25 L PET TJC) changed as the trial progressed. The 50:50 ratio of intervention to control was maintained in Grat-Tsatse, while in Dengolat the proportion in the intervention group declined over the course of the trial. In the other two areas, however, there was an increase in the proportion of children in the intervention group. Figure 4 shows the rates of diarrhoea in the control and intervention groups. The rate of reported diarrhoea declined dramatically over the first half of follow-up, becoming essentially zero by the end of the study.

The rates of dysentery are considerably smaller. Table 1 shows the period prevalence of diarrhoea and dysentery (period prevalence meaning that it had occurred within the fortnight prior to interview) over the whole trial period. There was no difference in the prevalence of diarrhoea (P = 0.320) or dysentery (P = 0.437) between the intervention and control groups, adjusting for age and area.

**Table 1:** Prevalence of diarrhoea and dysentery in the control and intervention groups.

Group	Period prevalence of diarrhoea (%)	Period prevalence of dysentery (%)	Number of observations
All children	1.1	0.2	22,396
Control (2 L PET bottle)	1.0	0.2	12,138
Intervention (25 L PET TJC)	1.1	0.3	10,258

## Discussion

In this work, the feasibility of scaling-up of the SODIS process in rural communities of the Tigray region in northern Ethiopia was tested. Microbiological evaluations of the large-volume containers under controlled and natural conditions showed that the daily dose accumulated in Tigray is sufficient to remove bacterial contamination. 25 L PET TJC were evaluated for microbiological inactivation in the laboratory to calculate the required UVA doses. Irradiance, temperature and water composition (turbidity, (bi)carbonates and transparent and coloured dissolved organic matter) were tested. In all the experiments, the UVA dose required for 3-log reduction of *E. coli* was lower than the minimum UVA daily dose received in Tigray (9411 ± 55 Wh/m^2^). The disinfection rate was proportional to the irradiance. This fact provides some level of assurance for users concerned about disinfection levels that might be achieved under conditions of varying irradiance such as partially cloudy days. In addition, the disinfection rate is enhanced by increased water temperatures caused by solar exposure (synergistic effect), ensuring disinfection even under varying field conditions.

As it might be expected, the required UVA dose for 25 L containers was higher than for 2 L bottles, exactly 1.2 times higher. In low-volume containers (2 L), differences in radiation distribution could be neglected due to the short optical path. In contrast, in large-volume containers, the kinetics of the process is affected not only by the average value of the incident radiation, but also by the homogeneity in the distribution of the radiation^30^. The presence of naturally occurring substances in low-volume containers may lead to external damages in bacteria. However, if these substances are optically active, such as coloured dissolved organic matter, iron, or solids, their role as attenuating factors prevails over their role as an external source of damage. In the case of iron, its permeation into the cells also contributes to the intracellular Fenton process, enhancing bacterial damage, and achieving shorter disinfection times than those expected for a solely optically active substance. Even in the case of plain water exposed to the sun in large-volume containers, the uniformity index of radiation is lower than 1 (exactly 0.94) due to the refractive index of water. This may explain why the required UVA dose in the present study was 1.2 times higher for 25 L PET than for 2 L PET bottles. Nevertheless, the required dose is still far below the minimum accumulated solar dose available in Tigray throughout the year. Consequently, it can be concluded that solar water disinfection in large-volume containers (25 L PET TJC) in Tigray seems feasible.

However, to confirm the success of the SODIS scaling up, these promising water quality laboratory results always should be corroborated by the health impact and user acceptability of the implementation in the field. Results from the HIA showed that the rates of dysentery were considerably smaller and rates of diarrhoea dramatically declined over the first half of the monitoring period, tending toward zero by the end of the study, suggesting a possible problem with data collection. In this study, the data suggest that diarrhoeal disease completely vanished, which is counter-intuitive. Prevalence fell from an initial 8.5% in week 1–2% at 12 weeks, and to less than 1% at 24 weeks. By week 36 it was effectively zero. There are two explanations for this. The first one is that diarrhoeal disease was completely eliminated over a wide geographical area by solar disinfection. The other more likely explanation is that adherence to reporting by care-givers of diarrhoeal disease declined sharply to zero due to courtesy bias or to study fatigue. Hopefully other researchers in this field may benefit by being forewarned of the challenges encountered in this study and take appropriate action to mitigate their effects. On the other hand,although the results of the health study do not show any difference in the impact of 25 L PET TJC compared with 2 L PET bottles, the low incidence of diarrhoea and the unlikely incidence levels reported towards the end of the study make any interpretation problematic. The main problem is the low incidence of diarrhoea, and especially of dysentery, meaning that most study participants are non-informative. The fact that the study found no difference between the treatments is not definitive from a statistical point of view because its power to detect a difference was handicapped by the low incidence. However, the microbiological evaluation does support similar water quality in both methods. In the literature, we can find several favourable Health Impact Assessment that support the efficacy of using low-volume SODIS systems in communities of low-income countries. For SODIS clinical trials, the odds ratio relates the odds of a disease event occurring in the test group to the odds of it happening in the control group. Over the last 25 years, reductions in the incidence of diarrhoea have been reported in the test group using a SODIS technology. For example, an odds ratio of 0.66 (95% CI 0.55–0.87) in children under 5 years of age in the Maasai community^15^ and an odds ratio of 0.12 (95% CI 0.02–0.65, P = 0.014) in children living in the Tamil Nadu region of southeast India^17^. In addition, other studies in peri-urban and rural communities in Kenya, Zimbabwe, and South Africa showed reduced incidence of dysentery in children under 5 years of age following the use of SODIS with odds ratios of 0.56 (95% CI 0.40–0.79, P < 0.001), 0.43 (95% CI 0.20–0.95, P = 0.036) and 0.35 (95% CI 0.17–0.76, P = 0.011), respectively^9,18,31^. The widely recognised efficacy of low-volume SODIS systems in the literature and similar water quality results in our laboratory for large- and low-volume systems support the potential for scalability. The acceptability of the new systems was unaffected probably due to the consideration of the habits and traditions in the Tigray region: the similar design of the standard opaque jerrycan to the 25 PET TJC helped to ensure the usability of the latter: the sustainable large-volume SODIS container implemented in Tigray.

## Methods

### Microbiological evaluation of the transparent jerrycan

Daily accumulated values of total incident radiation and temperature during the year were obtained using the solar calculator tool developed by Moreno-SanSegundo and Marugán^25^. It estimates both diffuse and direct irradiation at the Earth’s surface using the solar spectrum at the atmosphere’s surface where the air mass (AM) is zero (well-known as AM 0.0 solar spectrum). Extinction in the atmosphere, i. e. absorption and scattering due to cloud coverage, as well as other minor contributions from temperature or humidity were considered using the solar vector as a function of the latitude–longitude pair.

25 L PET TJC were supplied by Envases Soplados (Andújar, Spain) (Fig. 1). These containers were originally designed to store olive oil and so comply with Europe regulations (EU) Nº 10/2011, (EU) Nº 2023/2006 and (EU) Nº 1935/2004 concerning food safety, ensuring they were suitable for drinking water use. The container dimensions were: height 526 mm, base length 240 mm, base depth 263 mm; with an average wall thickness of 0.55 mm. Preliminary studies of the optical and mechanical properties of these containers showed that the UV-A transparency of the plastic material was adequate and that the resistance of the 25 L PET TJC to falls and impact was adequate to ensure its suitability to the harsh field conditions^26,32^. 70 TJC units were used for the experiments to evaluate the microbial quality of the water, and 1650 TJC units were transported to Ethiopia for distribution to the communities for the health impact assessment in the field.

Laboratory experiments on water disinfection were carried out using two different strains of the faecal indicator organism *E. coli*. Samples of *E. coli* K12 (CECT 4624, corresponding to ATCC 23631) were obtained from the Spanish Culture Collection (CECT). A wild *E. coli* strain was also isolated from the wastewater treatment plant at Rey Juan Carlos University (URJC, Móstoles, Spain). Fresh liquid cultures were prepared by inoculation in Luria–Bertani nutrient medium and incubation at 37 °C with rotary shaking for 24 h. To prepare the reaction media, 5 mL of the liquid culture were centrifuged at 3000 rpm for 15 min. Bacteria were separated from supernatant, rinsed again with 5 mL of sterile saline solution (NaCl 0.9%), and diluted into the experimental container to obtain the initial concentration. Viable *E. coli* colony forming units (CFU) in the samples taken during the experiments were quantified using a standard serial dilution method and plating in ENDO Agar (Merck KGaA, Darmstadt, Germany) with the colonies counted after incubation for 24 h at 37 °C.The detection limit of this method was 2 CFU/mL. Experiments under controlled conditions with a large-scale solar simulator at the URJC laboratory were carried out by inoculating wild *E. coli* at an initial concentration of 10^3^ CFU/mL into tap water after the addition of sodium thiosulfate to remove residual chlorine. Experiments under solar light at the Plataforma Solar de Almería (CIEMAT, Spain) were carried out using an initial concentration of 10^6^ CFU/mL of *E. coli* K12 in a ten-fold diluted well water (composition detailed in Table S2).

Microbiological inactivation was evaluated in terms of log reduction value (LRV) of the indicator microorganism *E. coli*. Assuming a typical field concentration of *E. coli* around 1–100 CFU/mL^33–35^, the required time to achieve 3-LRV by solar disinfection in the transparent jerrycans was estimated as a function of the irradiance, temperature and chemical composition of the water under simulated and natural sunlight. Values of times to achieve 3-LRV were rounded to the nearest 0.5 h multiple (error ± 0.25 h). If 3-LRV is not experimentally achieved, the time to achieve 3-LRV was estimated by extrapolation of the experimental curve. The disinfection profiles with the UVA dose under controlled and natural conditions can be found in Figs. S2 and S3, respectively. Experiments were performed in duplicate to ensure reproducibility in the bacterial quantification and obtain error bars in Figs. S2 and S3. Solar doses were calculated by multiplying the time by the irradiance and were compared with the lowest daily accumulated dose in the study area of the Tigray region in Ethiopia. Disinfection in large-volume containers would be feasible if the accumulated dose is higher than that required to achieve 3-LRV.

Experiments under controlled conditions in a large-scale solar simulator were carried out using a xenon lamp (Osram XBO 5000 W/H XL) with a temperature colour of 6000 K located on a customised reflector to ensure adequate homogeneous illumination of the water container. The emission spectrum of this lamp agrees adequately with the solar spectrum at the Earth’s surface in the UV range^36^. Irradiance at the surface of the container was measured by spectroradiometry using a calibrated StellarNet Spectrometer UVIS-25 (329–400 nm). The experiments were carried out at values of UVA irradiance of 16.6, 21.6, and 28.2 W/m^2^ and water temperatures of 25, 35, and 45 °C in agreement with the expected values during the exposure to natural sunlight. Different water compositions with up to 20 mg/L of dissolved organic carbon (DOC) added as TDOM 3.3 mg/L of humic acids added as CNOM, 100 mg/L (bi)carbonate, and 20 and 50 NTU of turbidity were tested, according to the maximum levels reported in Sub-Saharan water sources^33,34^ and SODIS guidance^12^. Some of these substances have been reported to behave as attenuating factors for radiation transport in large-volume containers^30^.

Impact on microbial water quality was also tested by exposing the transparent jerrycans to natural sunlight at the Plataforma Solar de Almería facility. These experiments were carried out on both sunny and cloudy days and under natural ranges of UVA irradiance (15.7–29.2 W/m^2^) and water temperature (23–45 °C). Experiments carried out at near sunrise or sunset presented low irradiance conditions and experiments close to solar noon offered high irradiance conditions. In addition, simultaneous disinfection in transparent jerrycans and 2 L PET bottles were performed to compare the dose required depending on the container volume. Irradiance at the surface of the container was measured using a Kipp & Zonen CUV-5 radiometer (280–400 nm). Irradiance measurements were normalised to the UVA range of the controlled conditions (329–400 nm).

From a microbiological perspective, this work focuses on bacterial inactivation by SODIS process and does not consider its effect on viruses and protozoa. However, recent studies show that viruses cannot be inactivated by SODIS in PET devices^37^, and that protozoa can only be thermally inactivated when the water achieves temperatures up to 40 °C^38^. Therefore, for both pathogens, the loss of radiation caused by the depth of the column water does not matter and, therefore, the size of the container is negligible.

### Health impact assessment

The overall aim of the health impact study was to show that solar disinfection of drinking water using the 25 L PET TJC was equivalent to that achieved in the standard 2 L PET bottles which are commonly used in SODIS. The specific objectives for the health impact assessment were confirmed only after satisfactory results were achieved in the monitoring of the water quality with the use of TJC under controlled conditions in the lab and under real sunlight pilot conditions, and these objectives were:

1. Assessment of the improvement in the health of children under 5 years old following the provision of solar disinfected drinking water at the point of use.
2. Assessment of the relationship between solar disinfected drinking water and selected health indicators.
3. Demonstration of the comparable effectiveness of SODIS using 25 L PET TJC as the intervention group and 2 L PET bottles as the control group.

The study was carried out in four rural communities in the Tigray Region in Northern Ethiopia. The region has a population of 4.3 million people of whom 80% live in rural zones^39^. The study sites were selected from the Northern, Eastern and Southern zones of Mekelle (the regional capital city of Tigray) as follows: Dengolat, 36 km South of Mekelle; Grat-Tsatse, 38 km North-East of Mekelle; Harena, 15 km North of Mekelle, and Tsawnet, 55 km North of Mekelle. The rationale for this selection was based on local information of prevalence of unsafe water sources, number of households, number of children under 5 years old, and the rates of diarrhoea measured in a baseline study. Background studies of the impact of diarrhoea, anaemia and internal parasitosis among pre-school children in the region can be found in previous studies^21,22^. An in-depth study of the sociological aspects of water in these communities can also be found elsewhere^40^. Ethical clearance for the study was obtained from the College of Health Sciences at Mekelle University (ERC 0844/2016) and the Royal College of Surgeons in Ireland (REC1400). The study was also registered at the Pan African Clinical Trial Registry (PACTR201710002676407).

Table 2 shows the characteristics of the children enrolled. Between the four sites, there were 1310 children under 5 years old in 988 households with a period prevalence of diarrhoea in the previous year of 13.5%. To compare the reduction of diarrhoea between the use of traditional 2 L PET bottles and the large-volume transparent jerrycan, half of the households were allocated to use 2 L PET bottles and the other half used 25 L PET TJC. Daily monitoring reports about diarrhoeal episodes of the 1310 children were collected.

**Table 2:** Characteristics of the case study sites.

Site	Coordinates	Unsafe Water Sources	Household	Children < 5	Diarrhoea baseline: 1-year period prevalenve (%)
Dengolat	13°28′56′′ N, 39°28′52′′ E	OP	292	368	10
Grat-Tsatse	13°34′56′′ N, 39°40′52′′ E	OP & S	313	427	10.5
Harena	13°33′0.88′′N, 39°32′24.60"E	OP & UW	206	265	8
Tsawnet	14°0′49.27′′ N, 39°27′23.39′′ E	OP	177	250	25.2

OP, open pond; S, stream; UW, unprotected well.

The methodology of the study is broadly similar to that used in previous studies of the implementation of SODIS using 2 L PET bottles in other African and Asian countries^18,31,41^. In this case, two 25 L PET TJC were distributed to each household in the intervention group. One of the 25 L PET TJC was filled with available water and was exposed to the sun all day while the other was kept in the dwelling and contained water disinfected on the previous day and available for use. The following day the roles of the containers were exchanged. The 25 L PET TJC kept in the house the day before was refilled with water and exposed to the sun and the disinfected water was used as storage and source water. At the end of the day, caregivers completed a data sheet developed by Gundry et al.^42^, which shows the incidence of diarrhoea of the child during the day. In the sheet, one happy and four sad faces correpsonded to the level of diarrhoea: the happy face crossed means absence of diarrhoea and from 1 to 4 sad faces crossed indicates the number of diarrhoeal episodes. The datasheet template can be found in the supplementary information (Fig. S4). Households in the control group were given two 2 L PET bottles for each child under 5 years of age and then followed a procedure identical to that of intervention group regarding SODIS exposure and storage of SODIS treated water.

### Ethical approval

All methods were carried out in accordance with relevant guidelines and regulations. Ethical clearance for the study was obtained from the College of Health Sciences at Mekelle University (ERC 0844/2016) and the Royal College of Surgeons in Ireland (REC1400). The study was also registered at the Pan African Clinical Trial Registry (PACTR201710002676407). Informed consent was obtained from all subjects and/or their legal guardian(s).

## Supplementary Information

Supplementary Information.

## Data Availability

The datasets generated and/or analysed during the current study are available in the Zenodo repository: https://doi.org/10.5281/zenodo.6656941.
