# Supplementary material for: Solar water disinfection in large-volume containers: from the laboratory to the field. A case study in Tigray, Ethiopia
Source: Sci Rep. 2022 Nov 7;12:18933. doi: 10.1038/s41598-022-23709-5 (PMC9640691; doi:10.1038/s41598-022-23709-5)
Supplement: Supplementary file 1 — Supplementary Information. [file 41598_2022_23709_MOESM1_ESM.pdf]

Supplementary Information

Solar water disinfection in large-volume containers: From the laboratory to the field. A case study in Tigray, Ethiopia.

Ángela García-Gil[1], *Rafael A. García-Muñoz*[1], *Azahara Martínez-García*[2], *Maria Inmaculada Polo-López*[2], *Araya Gebreyesus Wasihun*[3], *Mekonen Teferi*[4], *Tsehaye Asmelash*[5], *Ronan Conroy*[6], *Kevin G. McGuigan*[7], *Javier Marugán*[1,*]

[1] Department of Chemical and Environmental Technology (ESCET), Universidad Rey Juan Carlos, C / Tulipán s/n, 28933 Móstoles, Madrid, Spain.

[2] Plataforma Solar de Almería – CIEMAT, Carretera Senes, Km 4, 04200 Tabernas (Almería), Spain.

[3] College of Health Sciences, Department of Medical Microbiology and Immunology, Mekelle University, Tigray, Ethiopia.

[4] College of Natural and Computational Sciences, Department of Biology, Mekelle University, Tigray, Ethiopia.

[5] College of Health Sciences, Department of Medical Microbiology, Aksum University, Tigray, Ethiopia.

[6] Data Science Centre, Royal College of Surgeons in Ireland (RCSI), Dublin 2, Ireland.

[7] Dept. of Physiology and Medical Physics, Royal College of Surgeons in Ireland (RCSI), Dublin 2, Ireland.

*Corresponding author email address: javier.marugan@urjc.es

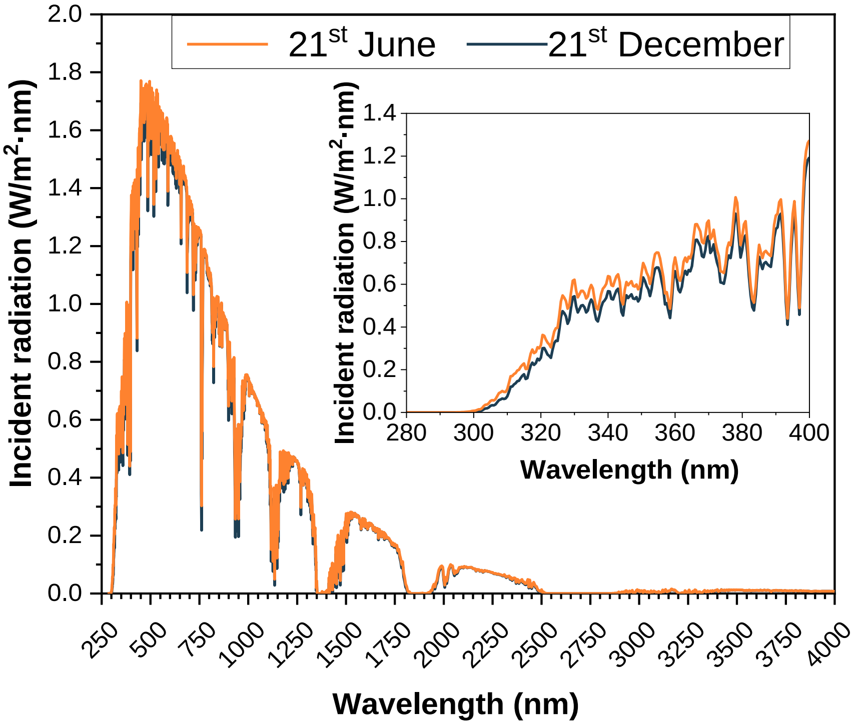

Fig. S1.A: Solar spectra in Tigray at successive solstices based on recorded historical (over the period 2011-2019).

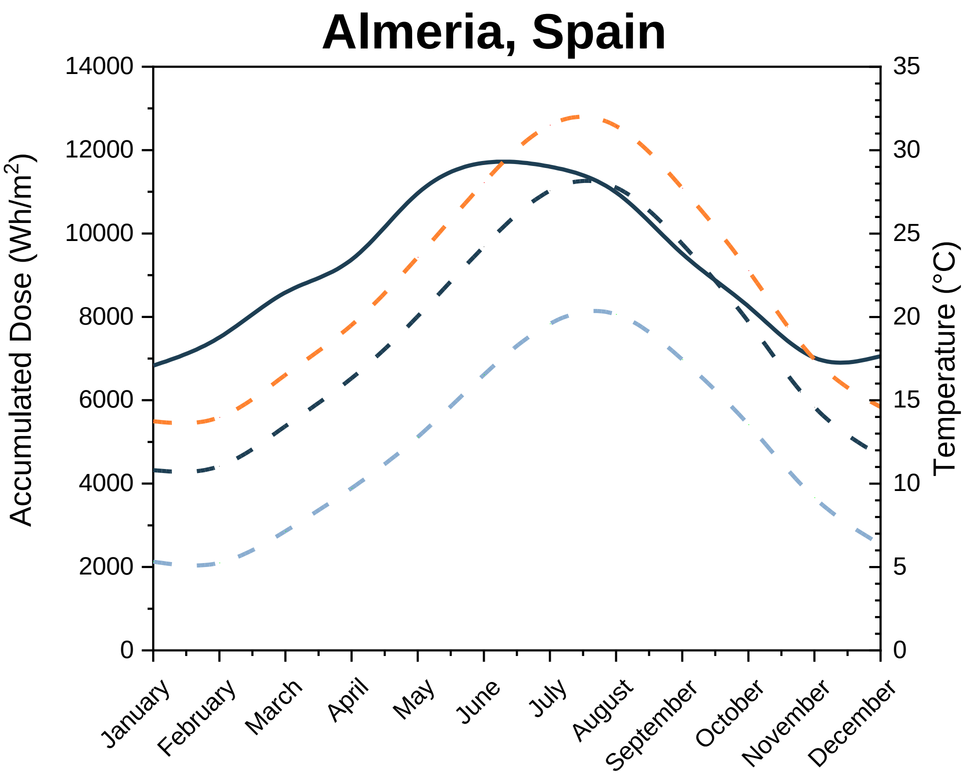

Fig. S1.B: Accumulated global dose in Almería (Spain) during the year. (solid line: accumulated global dose, dash line: maximum, average and minimum temperatures)

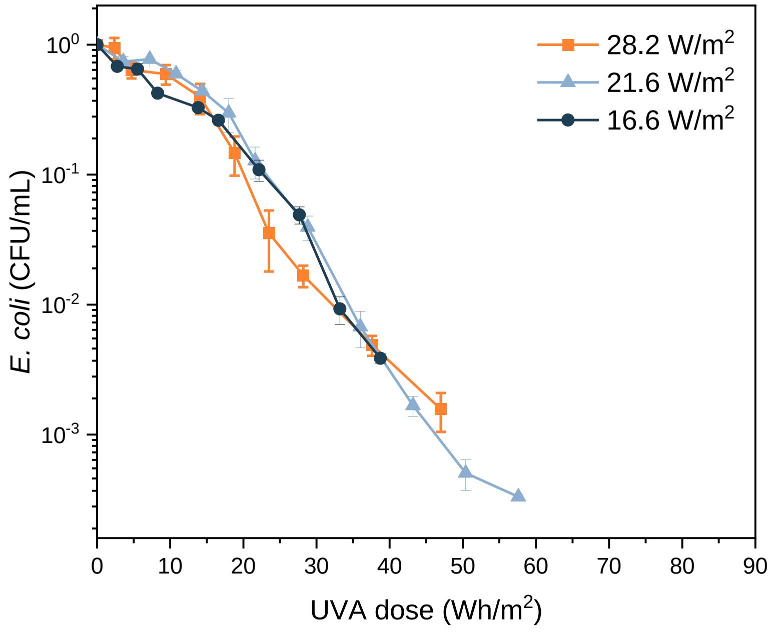

Fig. S2.A: *E. coli* inactivation profile with the UVA dose for different irradiances under controlled conditions for the 25 L PET TJC (Curves overlap since disinfection rate is normalised to the irradiance).

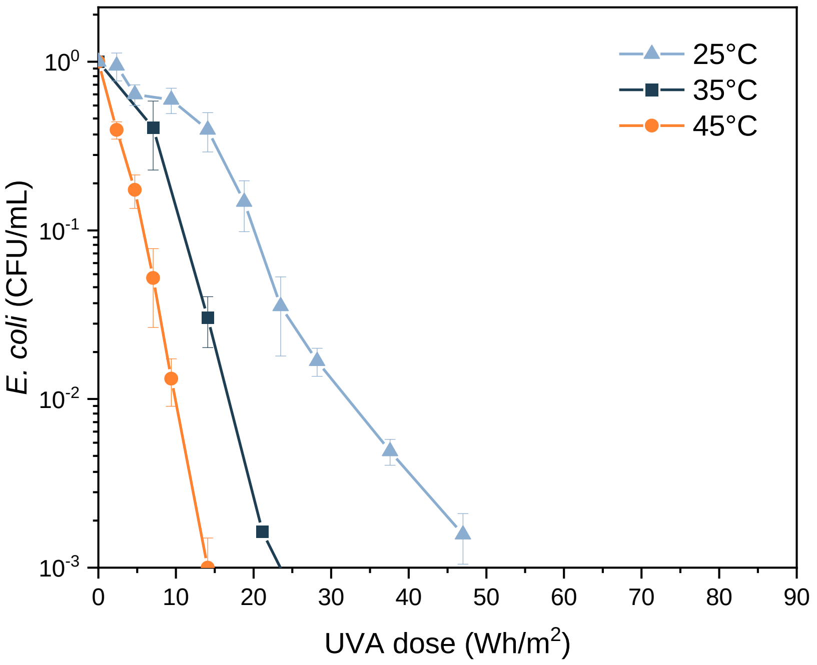

Fig. S2.B: *E. coli* inactivation profile with the UVA dose for different temperatures under controlled conditions of irradiance (28.2 W/m^2) for the 25 L PET TJC.

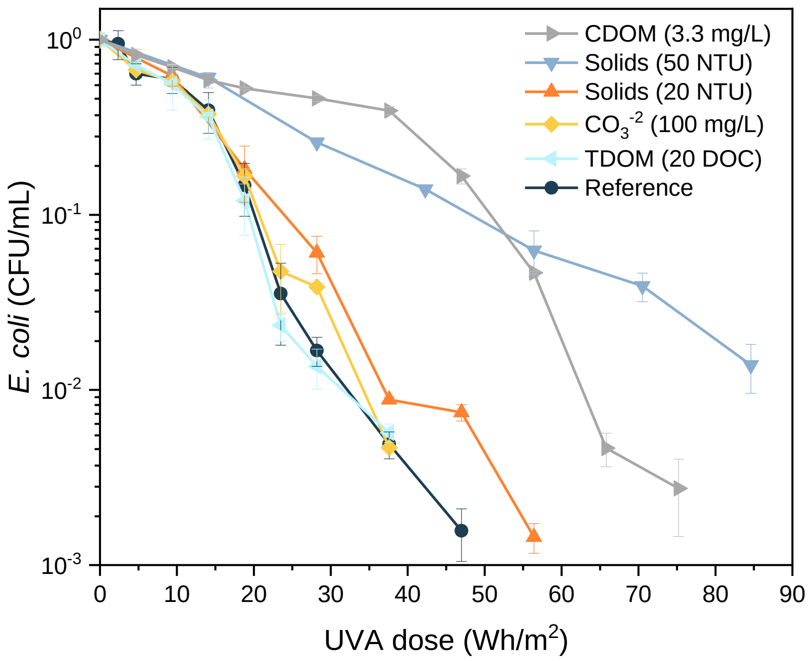

Fig. S2.C: *E. coli* inactivation profile with the UVA dose for different chemical water composition under controlled conditions of irradiance (28.2 W/m^2) and temperature (25°C) for the 25 L PET TJC.

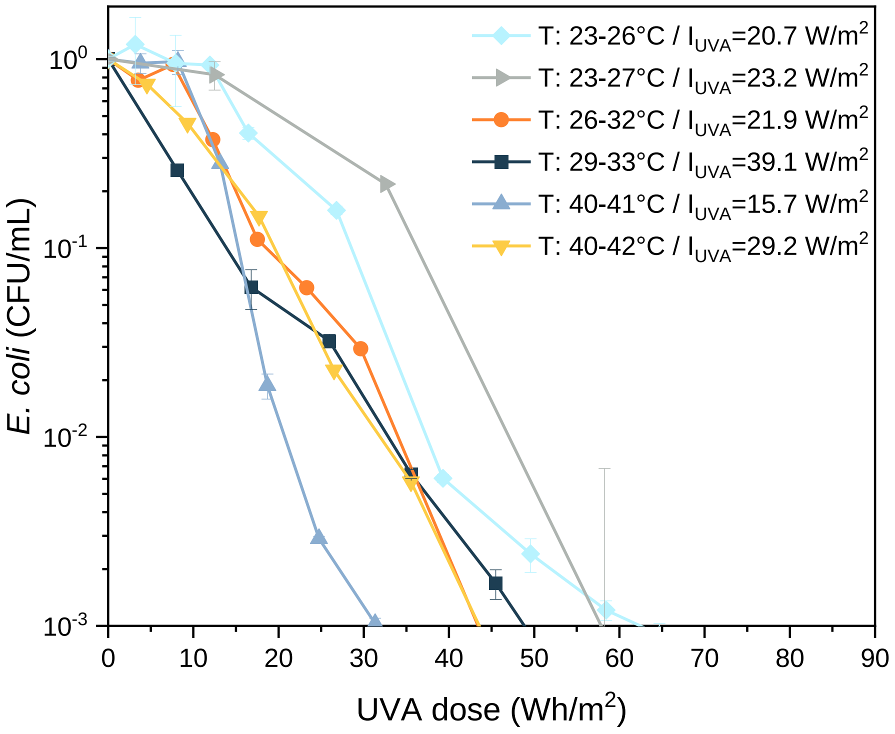

Fig. S3.A: *E. coli* inactivation profile with the UVA dose for diverse natural conditions.

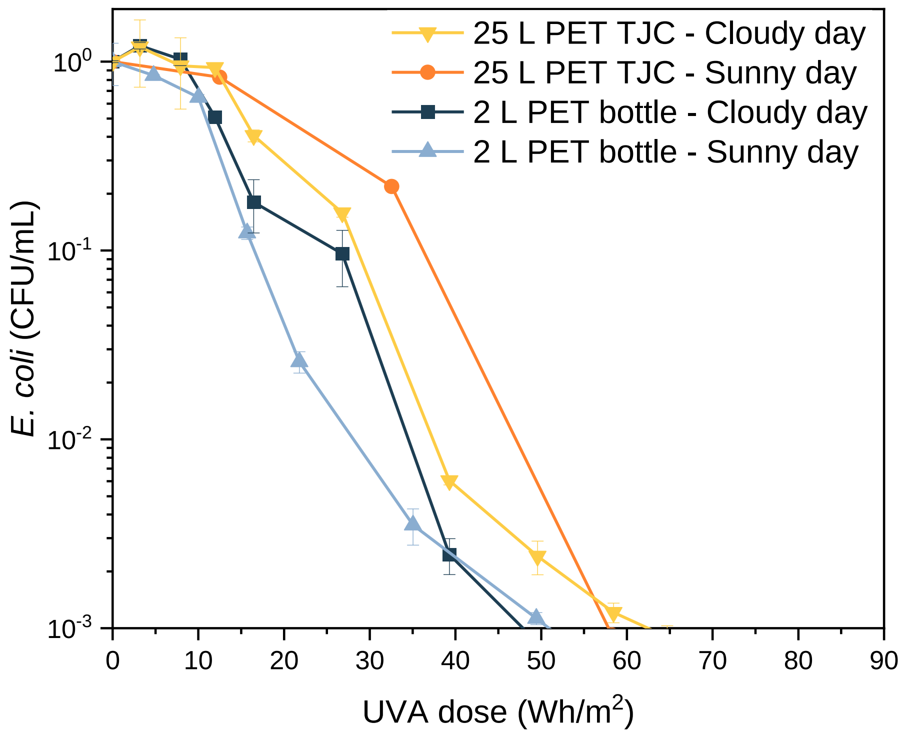

Fig. S3.B: Comparison of the *E. coli* inactivation profiles with the UVA dose in large-volume (25 L PET TJC) and small-volume (2 L PET bottles) containers under natural conditions

Table S1: UV and total solar irradiance at Tigray in solstices.

	Solstice of June	Solstice of December
UVB (280-329 nm) (W/m^2)	6.9	5.5
UVA (329-400 nm) (W/m^2)	51.7	47.3
Total (280-4000 nm) (W/m^2)	1071	1035

Table S2: Composition of well water used in experiments under real sunlight.

Ions (mg/L)	Concentration
Cl^-	38.15
NO_2^-	0.089
Br^-	0.262
NO^{3-}	1.000
$(PO_4)^{3-}$	0.848
$(SO_4)^{2-}$	22.75
Na^+	50.81
NH^{4+}	0.213
Ca^{2+}	1.045
K^+	9.460
Mg^{2+}	6.228
Conductivity (µS/cm)	364.5
Turbidity (NTU)	< 0.5 NTU

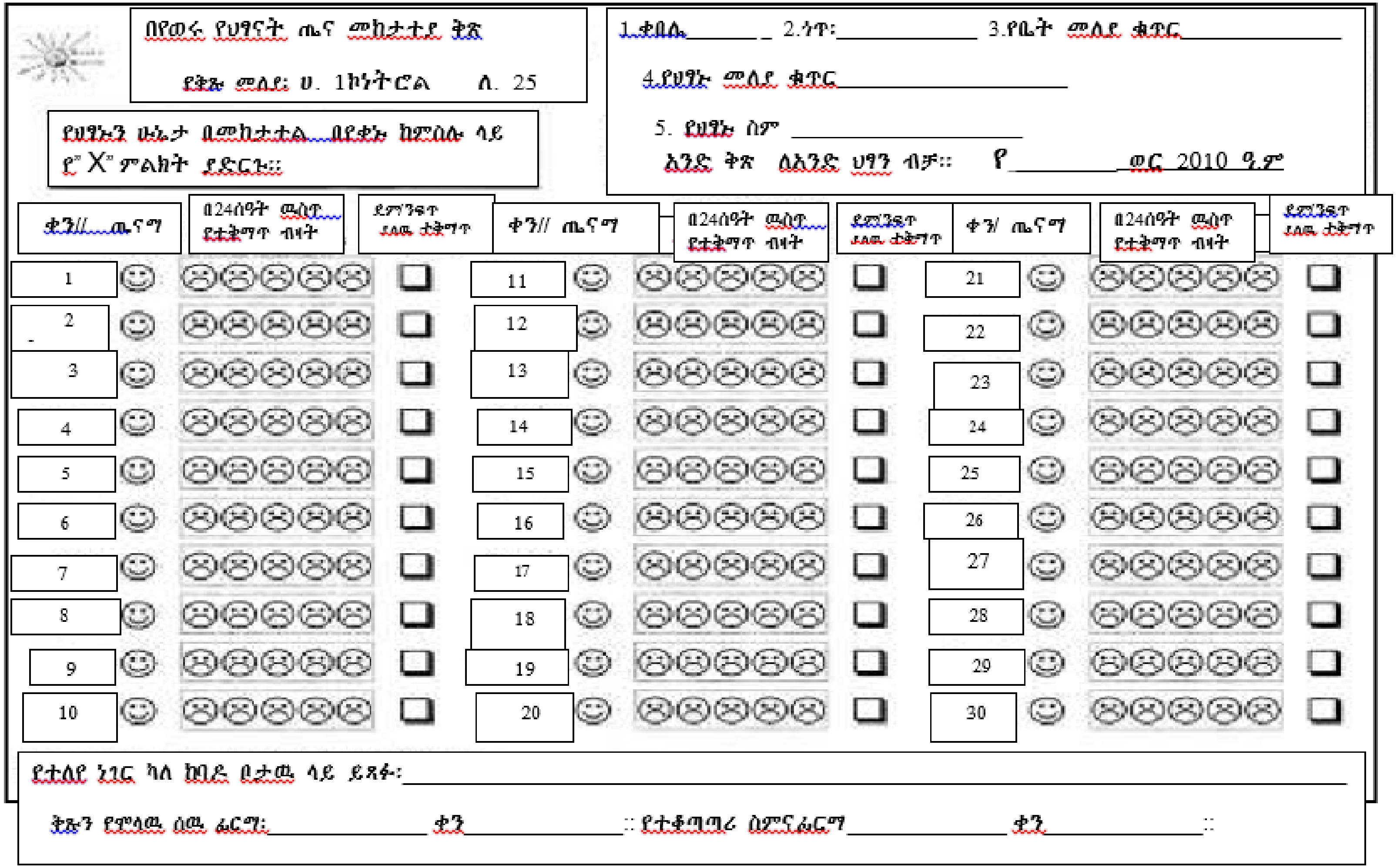

Fig. S4: Example of a monthly diarrhoeal diary (using fictitious names and addresses) for the monitoring of diarrhoeal episodes in the field
